# Go for it! Exercising makes you happy and strong

**DOI:** 10.37825/2239-9747.1019

**Published:** 2020-10-01

**Authors:** M Illario, V De Luca, A Cano, D Tramontano

**Affiliations:** 1 Department of Public Health; Research & Development Unit, Federico II University & Hospital, Naples, Italy; 2 Department of Pediatrics, Obstetrics and Gynecology, University of Valencia, Spain; 3 Department of Molecular Medicine and Medical Biotechnology, Federico II University, Naples, Italy; Fondazione GENS, Naples Italy

**Keywords:** physical activity, well-being, resilience

## Abstract

Despite it is generally recognized the beneficial role of physical activity, large portion of the population is physically inactive. Very alarmingly, the well-known gender gap in physical activity is constantly increasing. Several barriers obstacle women to perform physical activity although exercising would be of paramount importance for their health in particular during pregnancy and menopause. In addition to physical health benefits, physical activity may influence well-being and resilience, greatly impacting on quality of life.

Here we explore the relationship between physical activity resilience and well-being in a group of 1107 female residents in the Metropolitan area of Naples.

## I. INTRODUCTION

Insufficient physical activity is a leading risk factor for non-communicable diseases and can also negatively affect mental health and quality of life. WHO recognizes physical inactivity as a serious and growing public health problem and aims to reduce it by 10% by 2025 [1]. An analysis published in *The Lancet Global Health*, in 2018, found that more than a quarter of adults globally are insufficiently physically active [2].

Daily physical activity can help prevent cardiovascular diseases, heart disease and stroke, reduces blood pressure in those with high blood pressure levels [3,4]. Moreover, physical activity reduces body fat, strongly associated with high blood pressure, thus preventing and controlling diabetes and obesity [5,6]. By increasing muscle strength and endurance and improving flexibility and posture, regular exercise helps to prevent back pain [7]. Regular weight bearing exercise promotes bone formation and may prevent many forms of bone loss associated with aging like osteoporosis [8] Running and aerobic exercise delay the development of disability in older adults. [9–12]

Although, physical activity is vital to the health of both women and men, yet across most countries women are far less physically active compared to the male counterparts (global average of 31·7% for inactive women *vs* 23·4% for inactive men. According to WHO there are gender differences in the barriers to participating in physical activity [13–15]. In that, for women the lack of time and energy, due to caring responsibilities, lower socio-economic status, body image, gender stereotyping, and concerns about personal safety, are severe obstacle to being physically active. In addition, women have specificities, which are in part influenced by the hormonal changes that their body suffers along life. This systemic hormonal effect is so because, in addition to the genital tract and the breasts, various organs and systems in the body are a target for sexual hormones such as mainly, but not only, estrogens. This includes the bone, the vascular tree and the heart, or the central nervous system, among others [16,17]. Physical activity is important at all age, from childhood to old age, for women it is crucial at two times of life: pregnancy and menopause. Over the past 50 years research on physical activity and pregnancy has been supporting the promotion of moderate to vigorous prenatal physical activity for maternal and child health benefits. Besides healthier weight gain during pregnancy [18], it influences also gestational hypertension and diabetes [19, 20] Nevertheless, few women are active during pregnancy, and a vast majority decrease their activities or even quit exercising. A particular period in women’s life is defined by menopause, in which a decline in the hormonal production of the ovary occurs in a short time interval [21,22]. During menopausal transition together with hot flushes or sleep disturbances, there is a change in body architecture, due to increase in the abdominal circumference and accumulation of visceral fat, which impact on body image. Concurrently, metabolic dysregulation favors detrimental lipid changes, increase in blood pressure and in insulin resistance, all leading to increased risk of metabolic syndrome [23–25]. The result of the picture depicted above is an impoverishment of menopausal women quality of life. Hormonal treatment has been proposed as an optimal option to globally reduce the impact of the hormonal changes, but this is only taken by a lower proportion of women [26]. On the other hand, Physical Activity(PA) has been shown to enhance the quality of life among menopausal women, possibly because of its effect on neuroendocrine balance and the release of endogenous opioids, which lead to decreased vasomotor symptoms, improvement of quality of sleep and relief of musculoskeletal pain [27]. Moreover, as at all age, also during menopause, physical activity by improving self-esteem and reducing depression and anxiety, and helping to manage stress, increases subjective perceived well-being [28].

Subjective perceived well-being tells us how we perceive our lives are going, so the concept of well- being embraces a full array of factors from the type of environment we live in, to our interaction with other people to the endeavors we undertake to realize our aims [29–31]. Measure of subjective well-being is a meaningful outcome not only for each individual but also for the whole society [32]. In particular tracking well-being should be important for public policy considering the close relation of high well-being to key health outcomes such as lower rates of healthcare utilization, lower workplace absenteeism and better workplace performance, change in obesity status and new onset disease burden [33,34]. Resilience is a major component of well-being, and, although its definition has evolved over time, it is usually cast in terms of the ability of individuals to tackle life’s challenges, and to carry on and persevere in the face of adversity, even to the point of turning it into a development opportunity [35–37]. High resilience is associated, by and large, with high positive well-being and thus, when exploring health protection concepts, the resilience trait is ideally suited for the exploration of the inter-relationships between positive well-being, environmental day-by-day stress, and health.

Since the publication in 2009 of the Report of the Stiglitz-Sen-Fitoussi Commission for the measurement of economic performance and social progress, leisure activities are included as a key indicator in all major measures and indexes of subjective well-being, life satisfaction, and happiness [38–40]. The physical activity component of leisure, is significantly associated with pursuit and maintenance of personal health, health-related quality of life and psychological well-being and resilience facilitating improved coping with daily life stress [41–43]. Moreover, participation to leisure physical activity creates opportunities for socialization fighting social isolation and loneliness and their detrimental effect on physical and mental health [44,45].

In this article, we attempt to take a picture of a group of women resident in the Metropolitan area of Naples according to their physical activity, and we use subjective well-being and resilience score as a read out.

## 2. Methods

### 2.1. Basic Features

Within the framework of the European Innovation Partnership on Active and Healthy Ageing, Action 3-Getting to Optimize Aging Life Quality (GOAL) project, Fondazione GENS Onlus (a non-profit organization; Gene Environment Interaction Studies [46] developed an anonymous questionnaire to well-being, resilience, and perceived health.

The questionnaire used in this survey is anonymous and not anonymized. Anonymity refers to data collected from respondents who are completely unknown to anyone associated with the survey.

Only the respondent knows that he or she participated in the survey, and the survey researcher cannot identify, in any possible way, the participants. No one, including the investigator, can link an individual person to the responses.

The questionnaire we collected fulfills these requirements, in that it does not contain: Name and surname, address, ZIP code, ID or social security number, contact information of any kind (phone, or e-mail address). As consequence, individuals participating in this survey cannot be discerned in any way by anyone of the researchers involved. For these reasons, anonymous surveys do not require ethical approval.

In fact, in an anonymous survey, a written consent would have the paradoxical effect of compromising anonymity. Thus, the usual position in anonymous surveys is that a positive response from a respondent is, in itself, evidence of consent. Participation in the survey was voluntary. Trained GENS personnel gave all the necessary information regarding the scope and aims of the study to the individuals willing to participate in the survey. The anonymous questionnaire (paper and pen) was handed and explained to each participant, who was requested to fill the questionnaire and hand it back to GENS personnel on the spot. On request, GENS personnel assisted participants in filling the questionnaire.

In the present study, we analyze the responses of 1107 female subjects aged 18–93.

### 2.2. Questionnaire Structure

The anonymous questionnaire, in Italian, collected information covering socio-demographic and health-related data on relevant determinants of subjective well-being:

1. Demographic information: Age, schooling (no school, primary, secondary, high school, college), civil status (single, married, widow, divorced/separated), employment/work.
2. Subjective Self-reported psychological well-being (SPWB). Here we adopted a short form (PGWB-S) of the original psychological general well-being index [47], developed and validated in its Italian version by Grossi et al. in 2006 [48]. The PGWB-S questionnaire covers the following domains: Anxiety, Vitality (positive), Depressive Mood, Self-Control, Positive Well-Being, and Vitality (negative), assessed on a 0–5 Likert scale for the four weeks before the date of the survey. For brevity, we will refer to the results of the PGWB-S questionnaire as subjective self-reported psychological well-being (SPWB).
3. Resilience, measured according to the two-item Connor-Davidson resilience scale (CD-RISC2) on a 0–4 Likert scale [49]. In 2005, Connor and Davidson proposed an abbreviated version of their original CD-RISC to reduce administration time. The two items used for this scale are item 1(“Able to adapt to change”) and item 8 (“Tend to bounce back after illness or hardship”). Connor and Davidson deemed those items to be capable of “etymologically capturing the essence of resilience.” with the advantage to reduce administration time [49]. An Italian version of CD-RISC2 was not available, and an ad hoc translation was prepared adopting the conventional forward–backward procedure [50].
4. Physical activity. Participants had to indicate whether or not they regularly engage physical activity.
5. Diagnosed diseases. We considered the following list: diabetes, gastritis, anemia, depression, osteoporosis, migraine, anxiety, cardiovasacular diseases (heart failure, arrhythmias, ischemic heart diseases, myocardial infarction), hypertension, cancer, allergy, arthritis, obesity, low back pain, colitis, none. The above reported list of diseases was prepared according to the relevant Organization for Economic Co-operation and Development (OECD) and World Health Organization (WHO) reports [51,52].
6. Body Mass Index. Within the section related to perceived health status, participants indicated their weight and height. Body mass index (BMI), computed by dividing weight in kilograms by height in meters squared, was categorized according to WHO guidelines, underweight: BMI less than 18.5 kg/m2; normal weight: BMI 18.5–24.9 kg/m2 (reference category); overweight: BMI 25–29.9 kg/m2; obesity: BMI 30–40+ kg/m 2 [53]. All subjects were requested to indicate weight and height.

### Perceived Health Status (PHS)

PHS1 Participants were asked to rate their health status at the moment of the survey - “How do you feel your health at the moment” as: excellent, very good, good, decent, poor; and respect to a year before PHS2 “How do you feel your health in respect to one year ago” as worse, same, better.

### Statistical analysis

Descriptive statistics were computed for all the indicators analyzed. Student’s t-test was performed to evaluate differences between two groups while multiple group comparison was performed by Anova test followed by Bonferroni analysis.

## RESULTS

### Sample Description

The main characteristics of the sample population are outlined in Table 1. The sample consisted of 1107 females with average age of 50,84±17,32 years, whereas the range was 18–93 years, since we did not collect questionnaires from participants below 18 years of age. The demographic data reported by this female population are by and large in agreement with the data reported by the Italian Institute of Statistic (ISTAT) for the city of Naples [54].

### Physical activity engagement

We ask respondent whether they regularly exercise or practice a sport and at first we investigated possible the correlation of physical activity with several demographic, life style and health related variables. As it is shown in tab 2 physical activity directly correlate with SPWB, resilience education, relational network and perceived health status, while it inversely correlates with age, civil status, self-reported diagnosed disease (SRDD) and BMI, finally physical activity does not correlate with occupation.

### SPWB and resilience in women exercising and non-exercising

In this female population only 389 subjects (35.14%) regularly perform physical activity or practice a sport (from now on E) while 718 subjects (64,8%) does not (from now on NE).

In the attempt to get an insight in the different level of engagement in physical activity, we try to draw a profile of E and NE analyzing in details the variables that correlate with physical activity.

Among the variables SPWB and resilience correlate with physical activity. Resilience and SPWB together represent both a general subjective judgement on how life is going (SPWB) and the ability to cope with event of life (resilience).

The average SPWB scores of the E subjects and of the NE subjects are respectively 70,90±16,85 and 63,23±19,13 (p <0,0001). Although, these values pose both groups in the area of Moderate distress, NE are in the lower part, close to severe distress, while E are in the upper part close to No distress.

**Figure 1:**
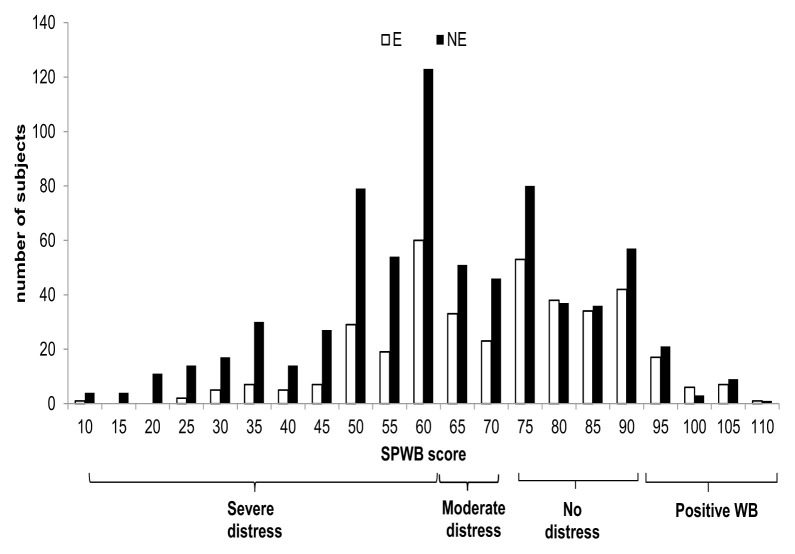
Distribution of E and NE according to SPWB score and categories.

The distribution of E and NE according to SPWB score is depicted in Fig 1 and it shows 52% of NE subjects are clustered in the area of severe distress while in the E group 34% falls in the same area.

In order to verify whether this difference could be due to the different contribution of the six dimensions composing of SPWB we analyzed each dimension separately in both E and NE subjects (tab 3).

We observed that the NE population scores lower than the E population in each of the six dimensions of SPWB. Of note, both E and NE scored consistently low in self-control and positive attitude. The average value of resilience scores in the E and NE population were respectively 6.131±1,510 and 5,584±1,883 (p<0.0001) in a range 0>8.

We also analyzed whether the two component of resilience “adapt to change “(item 1) and “Tend to bounce back after illness or hardship” (item2) differently contributed to the final score.

In that, it appears that both E and NE groups are more able to bounce back after illness of hardships than to adapt to change as they scored on average lower in item 1 than item 2 (NE 2,676±1.03 and 2.959±1.043 p<=0.0001; E 2.884±0,9275 and 3.247±0,8439 p<0,0001).

Finally, large part of this female population is pretty resilient as 72.5% of E and 66.4% of NE score 6 an above.

On the basis of the above reported results we analyzed the distribution of E and NE according to demographic characteristics, perceived health and relational network using SPWB and resilience as the read out of our analysis. The results are depicted in Tab 5, and show that the distribution of E and NE according to all the variables anal yzed is significantly different according to χ^2^. Moreover, in almost all the variables analyzed E scored higher than NE in both resilience and SPWB.

### Focusing on barriers

Age and presence of diseases are serious obstacles to physical activity for both man and women, however it is well recognized that women face also gender related barriers to stay physically active. Hence we decided to look at first at the relation of age and diseases with engagement in physical activity and afterwards at gender specific barriers in E and NE through the lens of caring responsibilities, body image and education.

### Age and physical activity

**Figure 3:**
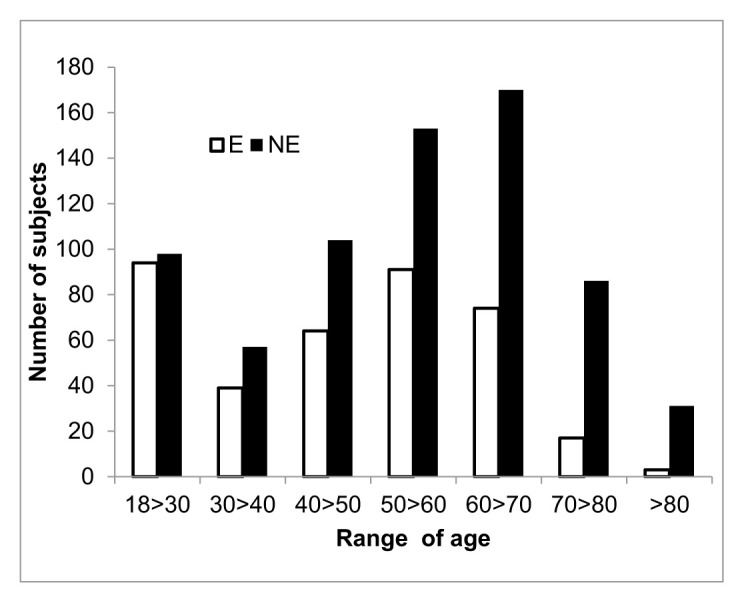
Distribution of E and NE according of different range of age.

The average age of E and NE groups is 47.02±16.63 and 52.91±17.34 (p<0.0001) respectively. Age distribution profile (χ^2^46,86, df 6 p< 0.0001) shows that E and NE substantially differ particularly in the range 50>60 and above year of age. (Fig 3)

We then analyzed SPWB and resilience according to the different range of age and two consideration appear interesting. On the one hand, the E group scored higher than the NE one independently of age range (tab 6), on the other that we did not observed significant variation of SPWB within each group with increase of age

**Figure 4:**
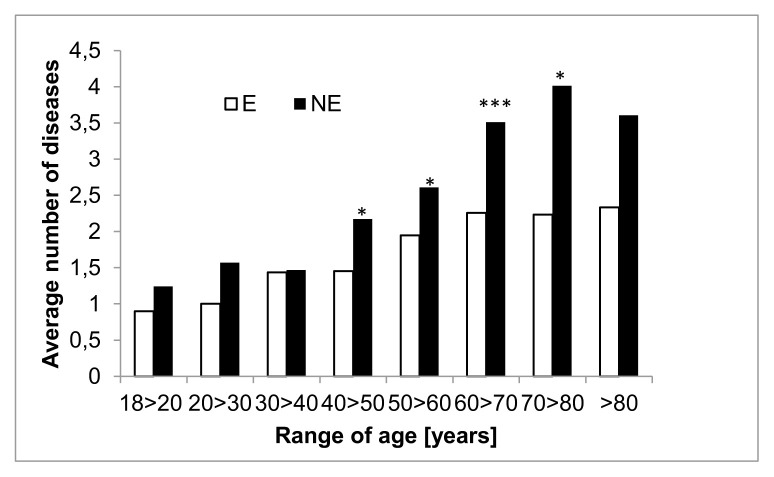
Distribution of E and NE age (years) according to number of SRDD

This latter is in line with the observation that in this female population, age does not correlate with SPWB and resilience (SPWB Pearson r −0,03622, CI 95%-0,09495 to 0,02277 p 0,2286 R square 0,001312; Resilience −0,01541 CI 95%-0,07427 to 0,04357, p value 0,6086, R, square 0,0002374). As the strong correlation of age with disease in E and NE group (E: Pearson r 0,2206, 95% CI 0,1232 to 0,3138, p value< 0,0001, R square 0,04866; NE: Pearson r 0,3341, 95% CI 0,2670 to 0,3981, p value< 0,0001, R square 0,1117), we examined the average number of disease according to different age range in E end NE group.(Fig 4)

As shown in Fig 4 the average number of disease started to be significantly different between E and NE starting from the 40>50 range of age, although already in the lower range of age we found significant difference in SPWB.

### Self-reported Diagnosed diseases (SRDD) and Resilience and SPWB

**Figure 5:**
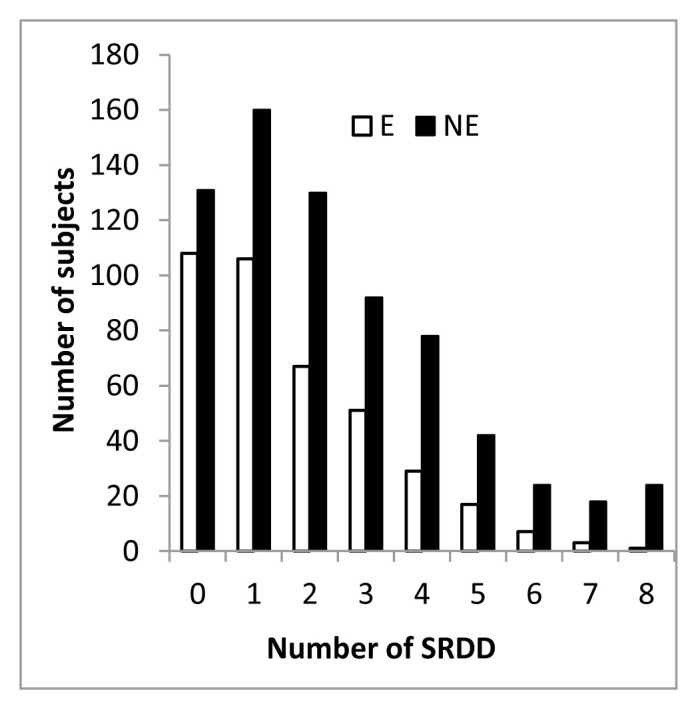
Distribution of E and NE for number of SRDD.

Because the strong correlation between diseases and physical activity we at first analyzed self-reported number of disease (from now on SRDD) in the two groups. On average NE subjects reported 2,684±2,5 while the E group 1,707±1,64 and the difference between E and NE SRDD is significantly different with a p value <0.0001. In addition, also the distribution of the number of self-reported diagnosed disease (SRDD) in the E and NE population was significantly different (X^2^43,95, df 9 p < 0,0001). It is of note that we compared only subjects reporting up to 8 diseases, and we found that in the NE population 20 subjects reported 9 up to 15 diseases, while in the E population only one subject reported 8 diseases (Fig 5).

Disease distribution show that the majority of E and NE subject are clustered in the range 0 >3, thus we examined in more details E and NE according 3 range of number of SRDD: 0, 1>3, 4>8. E and NE females reporting 0 diseases are superimposable for age (E 41.86±16.27 and NE 42.41±15.56) and resilience (E 6.06±1.45 and NE 5.81±1.78) while the two groups score differently in SPWB. In that, SPWB score of females exercising,73.76±15.35 is significant higher than that of not exercising counterpart (69.97±17.6, p<0.0322) and it falls in the range of No Distress. In addition, in the 0 diseases group 87% of E and 79% of NE perceived their health good, very good and excellent and 80% of E and NE consider their health same as the year before.

Within the group reporting from 1 to 3 diseases, in spite the average number of disease is similar between E and NE subjects, the E subjects score significantly higher in SPWB close to the range of No Distress, while the NE subjects are close to lower range of Moderate Distress. (Tab 7 a).

In the two groups the percentage of subjects reporting 1, 2 or 3 diseases does not substantially differ.

In 1>3 SRDD group 78% of E and 52% of NE perceive their health good, very good and excellent and 9% of E and 23% of NE considering their health worse than the year before.

Finally, E and NE groups reporting 4 to 8 diseases scored significantly different in age, average number of diseases with the E group being younger than the NE, having lower number of diseases, higher resilience and SPWB. However, E group in this case score in the lower range of Moderate Distress, while the NE one score frankly in the range of Severe Distress (Tab 7b).

According to the distrbution of SRDD, in the 4>8 range E cluster mainly between 4 and 5.

In the 4>8 SRDD group 49% of E and 72% of NE consider their health decent or poor and 23% of E and 40% NE considering their health worse than the year before.

**Figure 6:**
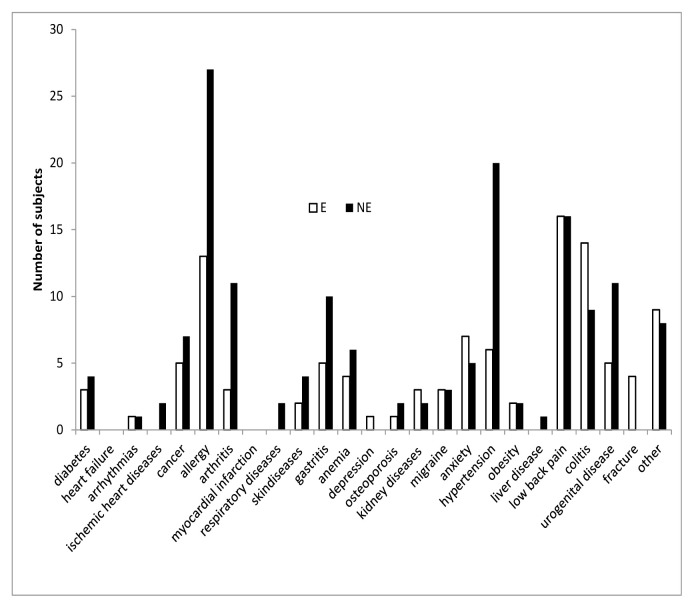
Distribution of E and NE reporting only one disease among the 25 disease option presented in the questionnaire. Moreover, we analyzed SPWB and resilience score in the subject reporting one disease according to each type of disease (tab 8). The number of subject in each group did not allow to make statistical evaluation for each diseases, however the overall SPWB and resilience scores reported by E group falls in the area of No Distress while the NE group is in the lower range of moderate distress (Table 8).

Finally, we try to verify whether a part from the number of disease, type of disease could be different between E and NE. To this end, we analyzed the distribution of E and NE reporting only one disease among the 25 disease option presented in the questionnaire. The result depicted in fig show that there are substantially no differences in the type of diseases reported by E and NE (Fig 6).

This result seems to suggest a role of physical activity in well-being and resilience independently from the type of disease reported, thus we analyzed, PSWB and resilience according to type of disease in the E and NE population independently of number of SRDD. As reported in table 9, once again E group scores significantly higher than the NE group in SPWB in the majority of the disease, analyzed and in some case also in the resilience score.

By and large all the data reported above suggest a relationship between engagement in physical activity and well-being and resilience apparently independent from both number and type of disease, as in all the setting tested E score substantially higher than NE.

### Housewife: the prototype caring responsibilities

Caring responsibilities are considered by women as one of the main obstacle to perform physical activity and/or practicing a sport. Housewife are the prototype of “caring responsibilities”, thus we examined the housewives group present in the female population under investigation. At first, it is of note that within the female population examined housewife average SPWB score is 62,41±20,43 which falls in the lower range of moderate distress. Housewife average SPWB score just above those unemployed (60,68±18,29) and lower than retired (65,45±17,88) and working females (68,35±17,93).

Among the housewife population only 19,43% reports to regularly exercise, against 42,35% of the “working” population.

We compare resilience and SPWB score in housewife and working females exercising and non-exercising (tab 10). Resilience is similar in E and NE housewives, while the E working population score higher in resilience than the NE working. In addition, resilience score of the E and NE “Working” population was higher than that of “Housewives” E and NE. On the other hand, E Housewife SPWB score was higher than the NE Housewife, and interestingly E Housewife scored almost as high as E “Working”, while NE Housewife SPWB score was significantly lower than that of NE “Working”.

### Education

Finally, we examined resilience and SPWB according to two range of education “Low” corresponding to elementary and junior high school, lasting eight year from 6 to 14 years age, and “High” corresponding to university degrees. Among the “Low” group only 13.35% of the subjects regularly exercise while the percentage rise to 49,73% in the “High” group (tab 11)

The “Low” and “High” group differ for age in both the E and NE subjects, but there is no age difference between E and NE subjects in the “Low” and ”High” education range with the “low” group being significantly older in both case. As for resilience, both E and NE in the “High” group report a resilience score higher than that of the E and NE “Low” group. Moreover, resilience score is not significantly different in the E and NE of the “Low” group, while the E “High” score significantly higher than the NE “High” group. Interestingly, we observed that E subjects in both the “Low” and the “High” group report a similar high SPWB score, higher than that reported by the NE corresponding group.

### BMI

Body image appear to be a barrier for women to exercise or practicing a sport. There is clear evidence that obesity is linked with poor body image, although not all obese persons suffer from this problem or are equally vulnerable [55]. According to BMI 28.21% of the population under investigation is overweight and 10.12% is obese. While 57% of the normal weight population does not exercise in the overweight and obese population the percentage rises to 75% and 84% respectively.

Resilience score in Obese E and NE is not significantly different, although Obese E score higher than Obese NE. On the other hand, both Overweight E and Normal Weight E score significantly higher in resilience than the NE counterpart. SPWB score of E subjects was significantly higher than that reported by the NE in the three categories. Moreover, while SPWB was significantly different within the NE of the three groups, NE Normal weight scoring higher than obese and over-weight, in the E subjects of the three groups SPWB score was basically the same (Tab 12).

### Quality of relationships and extent of social network

Quality of relationships and extent of social network are a key factors determining wellbeing and resilience attending gym or practicing a sport among other effect are good way to meet people to fight isolation, thus we investigated social network in E and NE. In both the E and NE “the number of people to count on in case of need” correlates directly with SPWB (E: Pearson r 0,2807, 95% confidence interval 0,1671 to 0,3869, P value (two-tailed) < 0,0001, R square 0,07878; NE Pearson r 0,2049, 95% confidence interval 0,1204 to 0,2864, P value (two-tailed) < 0,0001, R square 0,04198) and resilience (E: Pearson r 0, 0,2388, 95% confidence interval 0,1228 to 0,3139, P value (two-tailed) < 0,0001, R square 0,05704; NE: Pearson r 0,10600, 95% confidence interval 0,01954 to 0,1908, P value (two-tailed) < 0,0164, R square 0,01123). It is interesting to note that in the NE the correlation of resilience with number of people to count on in case of need, though significant, it is weaker that in the E.

We examined the distribution of the E and NE subjects according to range of people to count on in case of need, the result of Chi square analysis indicated THAT TH distribution is significantly different (χ^2^, df 14,39, 3; p<0,0024. Moreover, while the percentage of E and NE is similar in the 3>5 and 6>8 range (E 40% >NE 37% and E 18,8% >NE 19%), it substantially differs in the two extreme range. In particular, the percentage of NE subjects in the 0>2 range 21% almost double of 11% of E in the same range, conversely in the >9 range the percentage of E 30% versus 20% NE in the same group.

In both the E and NE group

Finally, we analyzed SPWB and resilience score according to the four range of people to count on. As shown in table 13, both SPWB resilience score increases with the increase of people to count on, however E scored almost always higher than NE in SPWB and resilience score.

## Discussion

In our work to investigate a group of women living in the metropolitan area of the city of Naples, performing and non performing physical activity in the attempt to define a profile of sedentary women. At first, to set the ground we studied the possible correlation of physical activity with demographic, individual behavioral and life style variables, and health status perception. Physical activity, correlate with self-reported diagnosed diseases, health perception, and BMI and also with civil status, education and social network. In addition, as physical activity correlates with both subjective self-reported psychological well-being (SPWB) and resilience. Psychological well-being is an integral part of an individual’s capacity to lead a fulfilling life, including the ability to form and maintain relationships, to study, work or pursue leisure interests, and to make day-to-day life decisions. Disturbances to an individual’s mental well-being can adversely compromise these capacities and choices, leading not only to diminished functioning at the individual level but also broader welfare losses at the household and societal level. Psychological well-being is influenced not only by individual characteristics or attributes, but also by the socioeconomic circumstances in which persons find themselves and the broader environment in which they live. With this in mind, we decide to use SPWB and resilience score as read out our investigation. According to SPWB average scores, the E group is close to the area of No Distress while the NE group in close to the moderate distress. It has been reported that, when depression component is included in wellbeing evaluation, women SPWB score decreases [56]. However, when we analyzed the sixth dimension of SPWB included, in our questionnaire which comprises depressive mood, we found that the self-control and positive attitude were the two dimension in which both E and NE female population living in the metropolitan area of the city of Naples reported on average the lower scores. We cannot exclude that the observation that on average this female population as a whole falls in the Moderate distress range, as it is also for the counterpart male population, is due to the difficult socio economic situation the city of Naples is going through particularly since the 2008 crisis [57]. In that, it is of note that using the same questionnaire, in the city of Milan women score, about fifteen point higher than the Neapolitan counterpart [58]. As for resilience, 64% of this female population appear to fall in the higher part of the resilience scale, moreover resilience appears to be less prone to dramatic variation. In a narrative fashion, this group of women, as the male population participating to this survey living in the city of Naples are “endogenously resilient” and they are abler to “endure” than to “change” [59]. Resilience is at the same time an individual resource to face difficulties but it also can be molded by life events. In particular, the ability of Neapolitan “bunch back after stressful events” could have its root in the long and difficult history of the city of Naples with different domination following one another, with different rules, language and culture. In addition, the peculiar position of the city that lay between Mount Vesuvius, a volcano, and Campi Flegrei the largest super-vulcano in Europe, without forgetting Marsili, a large active undersea volcano in the Tyrrhenian Sea, may have had a role in molding the ability of this people to “endure” to survive [59]. Only 35% of the women participating to this survey regularly exercising and according to our results, they report SPWB and resilience scores higher than those non exercising. In an attempt to trace a profile of the NE subjects, guided by the results of the correlation data, we analyzed in details the variables that correlate with physical exercise. In particular, we focused on the variable that are considered barriers for engagement in physical activity. As the inverse correlation of physical activity and age, we analyze age distribution of the of E and NE females. Not surprisingly the NE group is older than the E group. E subjects score significantly higher than NE in Resilience and SPWB at all range of age examined, while within E and NE group neither resilience and SPWB differ. Thus, apparently age is not a factor contributing to explaining different SPWB and resilience scores observed in E and NE. Presence of diseases are usually considered a critical barrier for physical activity, but it is also consistently found that perception of poor health is a significant barrier to exercise. In spite, interventions studies show that there are physical activity programs appropriate for basically all kind of diseases and disabilities, for most the fear remain that physical activity can worsen their health. In this light we examined both number and type of SRDD and subjective perception of health status in E and NE subjects. To this end, we examined this female population according to three range of diseases, 0, 1>3 and 4>8. The results obtained depict three different intriguing scenario in which physical activity might play different role: 1- 0 SRDD. Absence of diseases it is not sufficient to explain the SPWB difference between E and NE, this could a case of “pure physical activity effect”; 2- 1>3 SRDD. Equal number of diseases is not sufficient to explain the difference in SPWB between E and NE. The latter is strengthened by the observation that SPWB and resilience score differ in E and NE subjects reporting only 1 disease of the same type; 3- 4>8 SRDD Difference in number of diseases between E and NE could be sufficient to explain the difference in SPWB. However, when we analyzed single disease independently of total number of disease we observed that E scored basically always higher in SPWB than the NE subjects. When we focus on gender specific barriers like caring responsibilities (Housewife>Working), BMI and education (Low” level of education> “High” level of education) we almost always found that women physical active “feel better” (SPWB) and “stronger” (Resilience) than those with an inactive sedentary behavior.

Apparently our results suggest on the one hand that physical activity is a key element for women self-reported psychological well-being, on the other that lower socio-economic status, body image remain an obstacle to perform PA. Other factors, like built environment limit physical activity at a population level. Lack of green space, parks, walkable and bicycle lane, heavy traffic, all conjugated with air pollution makes very difficult to freely perform physical activity in most cities around the world [60,61]. On top of that, insufficient or inefficient public transportation and lack of public gyms or sport facilities are very often insurmountable obstacles for those living in suburban neighborhood. Attending gym or practicing a sport among other effect are good way to meet people to fight isolation and the quality and the extent of social network and personal relationship are key factors influencing SPWB [62–64]. Our data apparently support the positive role of relationship and PA on SPWB and resilience. In that, on the one hand in both E and NE subjects SPWB and resilience scores increase with increasing number of people to count on. On the other, E subjects score higher than NE subjects in both SPWB and resilience.

In conclusion, taken together the results of our work contribute with relevant data from a wide urban population in a Mediterranean environment to relationship positive between physical activity SPWB and resilience. Large improvement have been made in the comprehension of the molecular mechanisms supporting the benefit of physical activity in preventing or ameliorating the progression of diseases and those information are the bedrocks of the link between physical health and physical activity. However, apparently, the maintaining a good health and to prevent diseases is not a motivation strong enough for women to try to overcome obstacles and barriers. Possibly since those obstacle and barriers shape and condition women life far beyond jeopardizing their access to physical activity. Policies tackling gender gap in physical activity should be a priority not only for the substantial impact on overall population health, but primarily to warrant gender equality in job, opportunity and all aspects of life.

## Limitations

Study limitations and strengths. The present study had some strengths and limitations. Major strengths are the sample size and sociodemographic information to characterize the study sample; subjective psychological wellbeing evaluation included a range of wellbeing indicators more than simply life satisfaction [65,66] and also resilience evaluation. Though these results may have important implications for the well-being and health of women at all ages, this study has several limitations. Self-report questionnaires enable the collection of a large amount of quantitative data, and generalization of the findings is possible, if the sample is randomly collected. Nevertheless, we are aware of the limitations of using self-report questionnaires, whose main disadvantage might be the possibility of providing invalid or biased answers. In particular, respondents may not answer truthfully because of social desirability, acquiescent and non-acquiescent response bias, and clarity of the items [67]. However, some of the problems can be countered through the careful design and application of self-reporting measures. For example, response bias can be removed by ‘reversing’ half the questions on a questionnaire so that the variable is scored by positive responses on half the questions and negative responses on the other half, thus cancelling out any response bias. This is the approach applied in the SPWB questionnaire, where half of the question are reversed.

The cross-sectional design of the study limited our ability to infer direction of causality. A longitudinal design would better support the causal link between physical activity and well-being and resilience [68–70]. However, this is not always true. In this regard, Pek and Hoyle note that in recent years there has sometimes been a superficial use of longitudinal design, and this did not allow to overcome the weaknesses of cross-section design [71]. Moreover, this problem is associated with the difficulties that arise when taking ongoing measurements on the same sample in order to prepare a longitudinal design. We are aware that the results in the current study should be interpreted with caution, and future research is needed to give a definite response.

## Figures and Tables

**Table 1:** Characterics of the sample

Demografic data		
**Females**	1107	
**Age**	50,84±17,32	
**Civil Status**		%
Married	629	56,82
Divorced	49	4,43
Widow	83	7,50
Single	337	30,44
NA	9	0,81
**Education**		
Elementary	81	7,32
JHS	155	14,00
SHS/Diploma	463	41,82
University degree	382	34,51
NA	26	2,35
**Occupation**		
Working	429	38,75
Unemplojed	67	6,05
Housewife	283	25,56
Retired	165	14,91
Student	118	10,66
NA	45	4,07

**Table 2:** Correlations of physical activity and demographic variables., health, SPWB, resilience; ***=p<0.0001

	Pearson r	95% *CI*	P value	P value summary	R square
Age	−0,1623	−0,2191 to −0,1043	< 0,0001	***	0,02633
Civil status	−0,093	−0,1513 to −0,03401	0,002	**	0,00865
Ecucation	0,2836	0,2279 to 0,3376	< 0,0001	***	0,08046
Occupation	0,05389	−0,006299 to 0,1137	0,0792	ns	0,0029
SRDD*	−0,1927	−0,2488 to −0,1353	< 0,0001	***	0,03713
BMI	−0,1925	−0,2554 to −0,1280	< 0,0001	***	0,03706
PHS1**	0,2828	0,2255 to 0,3381	< 0,0001	***	0,07996
PHS2***	0,1837	0,1235 to 0,2427	< 0,0001	***	0,03376
Relational Network	0,09316	0,02327 to 0,1621	0,0091	**	0,00868
Resilience	0,147	0,08879 to 0,2041	< 0,0001	***	0,0216
PWB	0,1956	0,1383 to 0,2517	< 0,0001	***	0,03827
* Self-reported diagnosed diseases					
** Percieved health status at time of the survey					
***Percieved health status respect to a year before the survey					

**Table 3:** Comparison of the six dimensions of PWB, between E and NE subjects. Results are reported as mean ±SD. The value has been calculated by Student’s -test between the two groups.

	E	NE	
			p value
**Anxiety**	12.42±4.39	10.95±4.95	p<0.0001
**Vitality (posiive)**	12.79±3.63	11.15±4.33	p<0.0001
**Depressive mood**	12.15±3.70	11.14±4.21	p<0.0001
**Self-Control**	**10.69±4.45**	**9.565±4.73**	**p<0.0001**
**Positive attitude**	**10.47±3.09**	**9.284±4.19**	**p<0.0001**
**Vitality (negative)**	11.89±3.68	10.4±4.18	p<0.0001
	**p<0.0001**	**p<0.0001**	

**Table 5:** SPW and resilience in E and NR group according to demographic, perceived health and relational network.

	E	NE	E	NE	E	NE		E	NE	
					**Resilience**			**SPWB**		
**Civil Status**			%	%			p value			p value
single	149	188	38.3	26.18	6.10±1.52	5.57±1.56	**0.0018**	69±17.4	63.52±17.54	**0.0045**
widow	17	66	4.37	9.19	6.76±1.43	5.38±2.06	**0.0119**	77.47±19.07	57.63±24.89	**0.0032**
divorced	20	29	5.14	4.04	6.45±1.46	5.34±1.98	**0.0395**	68.3±16.61	56.83±17.85	**0.0276**
married	203	426	52.18	59.33	6.06±1.46	5.65±1.93	**0.0085**	72±16.15	64.56±18.81	**< 0.0001**
NA	0	9	0	1.25						
	**χ** **2 22.83, df 3**	**< 0.0001**								
**Education**										
elementary	10	71	2.57	9.88	5.7±1.63	5.21±1.92	NS	77.7±12.59	56.28±22.04	**0.0037**
JHS	19	136	4.88	18.94	5.52±2.03	5.16±2.24	NS	67.21±16.5	60.93±20.87	NS
SHS Degree	164	299	42.15	41.64	6.01±1.58	5.70±1.78	NS	68.38±17.83	63.41±18.33	**0.0050**
University	190	192	48.84	26.74	6.31±1.36	5.93±1.58	**0.0140**	72.73±15.8	66.92±17.1	**0.0007**
NA	10	16	2.57	2.22						
	**χ** **2 89.43, df 3**	**< 0.0001**								
**Occupation**										
unemplojed	54	67	13.88	9.33	5.07±1.94	5.8±1.75	NS	62.19±14.92	59.53±20.18	NS
student	27	40	6.94	5.57	6.16±1.35	5.97±1.29	NS	65.5±17.45	63.54±15.69	NS
housewife	55	227	14.13	31.61	5.69±1.80	5.21±2.12	NS	71.84±15.59	60.21±20.85	**<0.0001**
retired	50	115	12.85	16.016	6.32±1.31	5.70±1.78	**0.0299**	68.92±15.69	63.95±18.62	NS
working	185	242	47.55	33.7	6.35±1.34	5.81±1.70	**0.0004**	73.42±16.95	66.69±17.57	**<0.0001**
NA	18	27	4.62	3.76						
	**χ** **2 50.18, df 4**	**< 0.0001**								
**PSH1**										
Poor	7	69	1.79	9.61	6.43±0.53	4.29±2.58	**0.033**	55±19.18	42.1±19.15	NS
Decent	76	274	19.53	38.16	5.89±1.9	5.49±1.90	NS	63±16.44	60.88±17.67	NS
Good	154	210	39.58	29.24	6.11±1.25	5.94±1.48	NS	69.3±16.4	69.6±15.77	NS
Very Good	87	92	22.36	12.81	6.31±1.53	5.95±1.39	NS	75.87±14	67.6±17.76	**0.0007**
Excellent	35	24	8.99	3.34	6.4±1.47	5.87±1.51	NS	84.94±14.28	72.5±17.5	**0.004**
NA	30	49	7.71	6.82						
	**χ** **^2^** **87.96, df 5**	**< 0.0001**								
**PSH2**										
Worse	36	176	9.25	24.51	6.02±1.23	4.92±2.30	**0.0059**	61.89±16.99	51.89±20.16	**0.0059**
Same	251	391	64.52	54.45	6.10±1.55	5.78±1.60	**0.0124**	71.6±16.41	66.7±16.02	**0.0002**
Better	70	87	17.99	12.11	6.33±1.38	6.03±1.55	NS	72.6±16.52	69.16±18.59	NS
NA	32	64	8.22	8.91						
	**χ** **^2^** **41.36, df 3**	**< 0.0001**								
**Relational network**										
0>2	32	112	11.8	21.83	5.62±1.91	5.33±2	NS	65.09±16.15	57.78±17.13	**0.0463**
3>5	109	193	40.22	37.62	5.84±1.66	5.47±1.96	NS	68.13±17.05	60.6±18.33	**0.0005**
6>8	51	98	18.81	19.1	6.21±1.4	5.53±1.76	**0.0175**	70.92±16.37	66.52±18.79	NS
9>11	39	35	14.39	6.82	6.41±1.25	5.68±0.99	**0.0078**	73.97±16.51	61.66±16.55	**0.002**
>12	40	75	14.76	14.61	6.8±1.2	6±1.9	**0.0175**	81.18±15.22	71.32±20.1	**0.0077**
	**χ** **^2^** **14.39, df 3**	**<0.0024**								

**Table 6:** SPWB and resilience scores in E and NE distributed according to 7 different range of age.

	SPWB				Resilience	
Years	E	NE	p value	E	NE	p value
18>30	69,41±15,87	64,06±16,76	0,0252	6,043±1,509	5,918±1,207	NS
30>40	74,38±13,67	63,14±18,75	0.0018	6,231±1,739	5,14±2,3	0.0074
40>50	70,69±15.88	63,96±19,57	0.023	5,859±1,521	5,212±2,121	0.037
50>60	72,74±17,15	64,57±18,2	0,0006	6,22±1,356	5,889±1,738	NS
60>70	70,04±15,81	62,76±19,31	0.047	6,297±1,496	5,706±1,927	0.0196
70>80	72,59±19,18	58,24±16,61	0.0162	5,824±1,912	5,198±2,119	NS
>80	68,67±9,074	67,06±17,67	NA	6,333±1,528	5,839±1,695	NA

**Table 7a:** Comparison of age, resilience, SPWB and average SRDD in the E and NE subjects reporting 1 to 3 diagnosed diseases

			1>3		
	N° of subjects	Age	Resilience	SPWB	SRDD1
E	220	48.37±16.15	6.142±1.53	70.76±16.97	1.758±0.8132
NE	380	52.83±16.91	5.587±1.86	65.06±18.3	1.817±0.7945
		p<0.0018	p<0.0002	p<0.0002	NS

**Table 7b:** Comparison of age, resilience, SPWB and average SRDD in the E and NE subjects reporting 4>8 diagnosed diseases

			4>8		
	N° of subjects	Age	Resilience	SPWB	SRDD
E	54	51.17±16.61	6.056±1.57	65.31±15.24	4.75±0.977
NE	184	60.17±15.38	5.475±1.99	57.96±18.96	5.27±1.414
		p<0.0002	p<0.031	p<0.009	p<0.0212

**Table 8:** SPWB and resilience score in the subject reporting one disease according to various type of disease.

		E		NE			E		NE	
SRDD	N° of subjects	mean	N° of subjects	mean		N° of subjects	mean	N° of subjects	mean	
diabetes	3	79,33±21,36	4	68,75±8,32		3	5,333±2,3	4	6,5±1,3	
heart failure										
arrhythmias	1	103	1	48		1	6	1	3	
ischemic heart diseases			1	92				1	7	
cancer	5	72,6±20,45	7	51,29±16,55		5	7	7	6,429±1,72	
allergy	13	74,77±12,99	27	73,44±17,84		13	6,462±1,4	27	6,296±1	
arthritis	3	80,67±12,7	11	68±18,15		3	7,333±1,15	11	6,636±1,36	
myocardial infarction										
respiratory diseases			2	81				2	6±1,41	
skin diseases	2	69,5±26,16	4	46±28,37		2	6	4	5±2,16	
gastritis	5	69,4±14,42	10	78,8±10,68		5	6,4±1,14	10	4,9±2,08	
anemia	4	70,5±17,45	6	65,33±14,69		4	6,5±057	6	4,833±1,72	
depression	1	66				1	6			
osteoporosis	1	77				1	7			
kidney diseases	3	75,67±15,18	2	55±5,657		3	5,667±4,04	2	3,5±2,12	
migraine	3	64,67±20,31	3	80,67±9,452		3	6,667±0,57	3	6±1	
anxiety	7	57,71±27,49	4	61,5±10,66		7	6,143±1,3	4	3,75±0,5	
hypertension	6	64,83±17,65	21	67,24±16,45		6	6,5±1,3	21	5,619±1,65	
obesity	2	68±18,38	2	64,5±7,778		2	6±1,41	2	6,5±0,7	
liver disease			1	55				1	6	
low back pain	16	69,31±16,14	16	66,25±16,16		16	6,438±1,36	16	5,938±1,8	
colitis	14	74,07±12,66	8	61,38±13,77		14	6±1,46	8	6,5±0,75	
urogenital disease	5	91±6,892	11	64,73±23,73		5	6,8±,64	11	5,091±2,25	
fracture	4	93,5±9,147				4	6,75±1,89			
other	9	71,56±16,52	8	62,25±17,48		9	5,778±1,78	8	5,875±1,45	
**Total**	**107**	**74,65±10,78**	**149**	**65,56±11,67**	**p=0.0145**	**107**	**6,339±0,5**	**149**	**5,568±1,1**	**p=0.0076**

**Table 9:** SPWB and resilience scores according to type of disease in the E and NE population independently of number of SRDD.

		RESILIENCE					SPWB			
	E		NE			E		NE		
	mean	sd	mean	sd	p<value	mean	sd	mean	sd	p<value
CVD	6.304	1.428	5.529	2.174	NS	69.52	19.47	62.8	21.19	NS
Hypertension	6.468	1.396	5.61	1.929	**0.0050**	71.98	17.5	61.39	18.98	**0.0008**
Obesity	6.083	1.24	5.681	1.52	NS	70.33	13.38	57.15	17.6	**0.0189**
Diabetes	5.25	0.9574	5.711	1.487	NS	64	13.74	63.18	21.12	NS
Cancer	6.364	1.748	5.786	2.217	NS	64.91	18.61	60.75	19.38	NS
Depression	5.333	1.455	4.17	2.44	NS	64.78	11.09	43.17	16.9	**0.0001**
Anxiety	5.34	1.797	5.389	2.073	NS	61.13	17.65	53.98	16.24	**0.0153**
Osteoporosis	6.143	1.693	5.579	1.913	NS	70.18	16.16	61.82	17.87	**0.0232**
Migraine	6.034	1.401	5.145	1.626	**0.0146**	61.34	14.08	62.56	18.49	NS
Anemia	6.44	1.044	5.652	1.649	**0.0342**	73.36	15.42	63.52	19.22	**0.0311**
Allergy	6.4	1.367	5.879	1.529	**0.0209**	72.62	14.44	64.95	17.57	**0.0027**
Artrosis	5.976	1.917	5.853	1.711	NS	69.44	15.4	61.19	18.48	**0.012**
Low back pain	6.197	1.317	5.698	1.932	**0.0428**	68.01	14	62.23	19.12	**0.0196**
Colites	5.859	1.562	5.452	1.891	NS	68.17	14.97	59.35	15.4	**0.0006**
Gastrites	6.146	1.798	5.318	1.91	**0.0155**	69.4	15.85	63.72	15.24	**0.0451**
**mean**	**6.022**	**0.409**	**5.497**	**0.4184**	**0.0017**	**67.94**	**3.872**	**60.12**	**5.446**	**0.0001**

**Table 10:** Age Resilience and SPWB in Housewife and Working subjects in E and NE groups.

		Housewife	Working	*p* value
**AGE**	**E**	55.94±1.538	49.26±0.842	0.0002
	**NE**	59.92 ± 0.888	48.97 ± 0.737	<0.0001
	*p* value	0,0459	NS	
**Resilience**	**E**	5.691±1.804	6.353±1.343	0.0035
	**NE**	5.189±2.152	5.829±1.658	<0.0003
	*p* value	NS	<0.0005	
**SPWB**	**E**	71.84±15.59	73.36±16.98	NS
	**NE**	60.14±20.83	66.57±17.49	<0.0003
	*p* value	<0.0001	<0.0001	

**Table 11:** Comparison of SPWB and resilience scores in E and NE subjects according to education: Low=elementary/Junior High School; High= University degree.

		EDUCATION		
		LOW	HIGH	*p* value
**Age**	**E**	55.62±2.849	48.77±1.028	0.0173
	**NE**	62.73±1.015	48.93±1.102	<0.0001
	*p* value	0.916	NS	
**Resilience**	**E**	5.686±1.778	6.311±1.362	0.0187
	**NE**	5.128±2.151	5.938±1.581	<0.0001
	*p* value	NS	<0.0140	
**SPWB**	**E**	72.74±16.16	72.73±15.89	NS
	**NE**	59.88±21.16	66.92±17.1	<0.0002
	*p* value	<0.0007	<0.0007	

**Table 12:** Comparison of SPWB and resilience scores in E and NE subjects according to BMI

		Obese	Overweight	Normal weight	*p* value
**Resilience**	**E**	6±1.71	6.147±1.519	6.284±1.475	NS
	**NE**	5.64±2.038	5.517±1.889	5.83±1.644	NS
	*p* value	NS	<0.0144	<0.0014	
**SPWB**	**E**	70.62±13.38	71.94±13.71	71.07±16.9	NS
	**NE**	59.24±19.19	60.26±19.19	67.26±17.57	<0.0001
	*p* value	<0.0439	<0.0001	<0.0144	

**Table 13:** SPWB and resilience scores in E and NE subjects according to number of people to count on in case of need.

		How many people you can count on in case of need				
		0>2	3>5	6>8	>9	p value
SPWB	E	65,09±16.15	68,13±17.05	71,82±16.37	77,62±16.17	p<0,0001
	NE	57,78±18.62	60,6±18.33	65,17±18.79	68,25±19.49	p<0,0001
p value		0,0463	0,0005	0.0275	0,0006	
Resilience	E	5,625±1.91	5,844±1.66	6,216±1.4	6,608±1.23	p<0,0001
	NE	5,339±2	5,477±1.96	5,531±1.76	5,9±1.67	p<0,0001
p value		NS	NS	0,0175	0,0017	

## References

[b1-tmj-23-04-092] 1 https://www.who.int/news-room/factsheets/detail/physical-activity

[2] Guthold R, Stevens GA, Riley LM, Bull FC (2018). Worldwide trends in insufficient physical activity from 2001 to 2016: a pooled analysis of 358 population-based surveys with 1·9 million participants [published correction appears in Lancet Glob Health. 2019 Jan;7(1): e36]. Lancet Glob Health.

[3] Warburton DER, Bredin SSD (2017). Health benefits of physical activity: a systematic review of current systematic reviews. Curr Opin Cardiol.

[4] Myers J, McAuley P, Lavie CJ, Despres JP, Arena R, Kokkinos P (2015). Physical activity and cardiorespiratory fitness as major markers of cardiovascular risk:their independent and interwoven importance to health status. Prog Cardiovasc Dis.

[5] Stanford KI, Middelbeek RJ, Goodyear LJ (2015). Exercise Effects on White Adipose Tissue: Beiging and Metabolic Adaptations. Diabetes.

[6] Hall JE, do Carmo JM, da Silva AA, Wang Z, Hall ME (2015). Obesity-induced hypertension: interaction of neurohumoral and renal mechanisms. Circ Res.

[7] Hendrick P, Milosavljevic S, Hale L, Hurley DA, McDonough S, Ryan B, Baxter GD (2011). The relationship between physical activity and low back pain outcomes: asystematic review of observational studies. Eur Spine J.

[8] Pinheiro MB, Oliveira J, Bauman A, Fairhall N, Kwok W, Sherrington C (2020). Evidence on physical activity and osteoporosis prevention for people aged 65+ years: a systematic review to inform the WHO guidelines on physical activity and sedentary behaviour. Int J Behav Nutr Phys Act.

[9] Shin CN, Lee YS, Belyea M (2018). Physical activity, benefits, and barriers across the aging continuum. Appl Nurs Res.

[10] Hupin D, Roche F, Gremeaux V, Chatard JC, Oriol M, Gaspoz JM, Barthélémy JC, Edouard P (2015). Even a low-dose of moderate-to-vigorous physical activity reduces mortality by 22% in adults aged ≥60 years: a systematic review and meta-analysis. Br J Sports Med.

[11] McPhee JS, French DP, Jackson D, Nazroo J, Pendleton N, Degens H (2016). Physical activity in older age: perspectives for healthy ageing and frailty. Biogerontology.

[12] Yeolekar ME, Sukumaran S (2014). Frailty Syndrome: A Review. J Assoc Physicians India.

[b13-tmj-23-04-092] 13 https://www.who.int/dietphysicalactivity/factsheet_women/en/

[14] The Lancet Public Health (2019). Time to tackle the physical activity gender gap. Lancet Public Health.

[15] Mayo X, Liguori G, Iglesias-Soler E (2019). The active living gender’s gap challenge: 2013–2017. Eurobarometers physical inactivity data show constant higher prevalence in women with no progress towards global reduction goals. BMC Public Health.

[16] Jia M, Dahlman-Wright K, Gustafsson JÅ (2015). Estrogen receptor alpha and beta in health and disease. Best Pract Res Clin Endocrinol Metab.

[17] Eyster KM (2016). The Estrogen Receptors: An Overview from Different Perspectives. Methods Mol Biol.

[18] Domingues (2020). Trials (2015) 16:227 DOI 10.1186/s13063-015-0749-3 physical activity reduces rate of preterm births. Int J Environ Res Public Health.

[19] Thompson EL, Vamos CA, Daley EM (2017). Physical activity during pregnancy and the role of theory in promoting positive behavior change: A systematic review. J Sport Health Sci.

[20] Krzepota J, Sadowska D, Biernat E (2018). Relationships between Physical Activity and Quality of Life in Pregnant Women in the Second and Third Trimester. Int J Environ Res Public Health.

[21] Cano A (2016). Physical activity and healthy aging Menopause.

[22] Davis SR, Lambrinoudaki I, Lumsden M, Mishra GD, Pal L, Rees M, Santoro N, Simoncini T (2015). Menopause. Nat Rev Dis Primers.

[23] Janssen I, Powell LH, Crawford S, Lasley B, Sutton-Tyrrell K (2008). Menopause and the metabolic syndrome: the Study of Women’s Health Across the Nation. Arch Intern Med.

[24] Shea KL, Gavin KM, Melanson EL, Gibbons E, Stavros A, Wolfe P, Kittelson JM, Vondracek SF, Schwartz RS, Wierman ME, Kohrt WM (2015). Body composition and bone mineral density after ovarian hormone suppression with or without estradiol treatment. Menopause.

[25] Marlatt KL, Redman LM, Beyl RA, Smith SR, Champagne CM, Yi F, Lovejoy JC (2020). Racial differences in body composition and cardiometabolic risk during the menopause transition: a prospective, observational cohort study. Am J Obstet Gynecol.

[26] Crawford SL, Crandall CJ, Derby CA, El Khoudary SR, Waetjen LE, Fischer M, Joffe H (2018). Menopausal hormone therapy trends before versus after 2002: impact of the Women’s Health Initiative Study Results. Menopause.

[27] Sternfeld B, Dugan S (2011). Physical activity and health during the menopausal transition. Obstet Gynecol Clin North Am.

[28] El Hajj A, Wardy N, Haidar S, Bourgi D, Haddad ME, Chammas DE, El Osta N, Rabbaa Khabbaz L, Papazian T (2020). Menopausal symptoms, physical activity level and quality of life of women living in the Mediterranean region. PLoS One.

[29] Ryff CD (2014). Psychological well-being revisited: advances in the science and practice of Eudaimonia. Psychother Psychosom.

[30] Ruggeri K, Garcia-Garzon E, Maguire Á, Matz S, Huppert FA (2020). Well-being is more than happiness and life satisfaction: a multidimensional analysis of 21 countries. Health Qual Life Outcomes.

[31] Tay L, Kuykendall L, Diener E (2015). Satisfaction and happiness – The bright side of quality of life. Global Handbook of Quality of Life.

[32] Diener E;, Oishi S, Lucas RE (2015). National accounts of subjective well-being. Am Psychol.

[33] Steptoe A, Deaton AA, Stone (2015). Subjective wellbeing, health, and ageing. Lancet.

[34] Huppert FA (2009). A New Approach to Reducing Disorder and Improving Well-Being. Perspect Psychol Sci.

[35] Earvolino-Ramirez M (2007). Resilience: A Concept Analysis. Nursing Forum.

[36] Hornor G (2017). Resilience. Journal of Pediatric Health Care.

[37] Fletcher D, Sarkar M (2013). Psychological resilience: A review and critique of definitions, concepts, and theory. European Psychologist.

[38] Stiglitz J, Sen AK, Fitoussi JP (2009). Report by the Commission on the Measurement of Economic Performance and Social Progress Document de Travail de L’Observatoire Français des Conjonctures Économiques (OFCE).

[39] Brajsa-Zganec A, Merkas M, Sverko I (2011). Quality of Life and Leisure Activities: How Do Leisure Activities Contribute to Subjective Well-Being?. Soc Indic Res.

[40] Newman DB, Tay L, Diener E (2014). Leisure and subjective well-being: A model of psychological mechanisms as mediating factors. Journal of Happiness Studies.

[41] Netz Y, Wu MJ, Becker BJ, Tenenbaum G (2005). Physical activity and psychological well-being in advanced age: a meta-analysis of intervention studies. Psychol Aging.

[42] Peralta M, Martins J, Gómez Chávez F, Cortés Almanzar P, Marques A (2018). Self-rated wellbeing and physical activity associations in European older adults. Eur J Sport Sci.

[43] Kekäläinen T, Freund AM, Sipilä S, Kokko K Cross-Sectional and Longitudinal Associations between Leisure Time Physical Activity, Mental Well-Being and Subjective Health in Middle Adulthood. Applied Research in Quality of Life.

[44] Cacioppo JT, Cacioppo S (2014). Social Relationships and Health: The Toxic Effects of Perceived Social Isolation. Soc Personal Psychol Compass.

[45] Toepoel (2013). Ageing, Leisure, and Social Connectedness: How could Leisure Help Reduce Social Isolation of Older People?. Social Indicators Research.

[b46-tmj-23-04-092] 46 http://gensstudy.org/

[47] Chassany O, Dimenäs E, Dubois D, Albert Wu, Dupuy H (2004). The Psychological General Well-Being Index (PGWBI) Manual User.

[48] Grossi E, Groth N, Mosconi P (2006). “Development and validation of the short version of the Psychological General Well-Being Index (PGWB-S)”. Health and Quality of Life Outcomes.

[49] Vaishnavi S, Connor K, Davidson JR (2007). “An abbreviated version of the Connor-Davidson Resilience Scale (CD-RISC), the CD-RISC2: psychometric properties and applications in psychopharmacological trials””. Psychiatry Research.

[50] Brislin RW, Lonner W, Berry J (1986). The wording and translation of research instruments. Field Methods in Cross-Cultural Research.

[51] State of Health in the EU OECD/European Observatory on Health Systems and Policies.

[52] Non-Communicable Diseases Country Profiles 2018 Italy.

[53] BMI WHO (1995). World Health Organization (WHO) Expert Committee on Physical Status. The use and interpretation of anthropometry. Report of a World Health Organization Expert Committee.

[b54-tmj-23-04-092] 54(https://2019/), /28910,8909

[55] Schwartz MB, Brownell KD (2004). Obesity and body image. Body Image.

[56] Senik C (2015). Gender Gaps in Subjective Wellbeing European Commission—Directorate-General for Justice.

[57] Rapacciuolo A, Perrone Filardi P, Cuomo R, Mauriello V, Quarto M, Kisslinger A, Savarese G, Illario M, Tramontano D (2016). The Impact of Social and Cultural Engagement and Dieting on Well-Being and Resilience in a Group of Residents in the Metropolitan Area of Naples Volume 2016.

[58] Grossi E, Compare A, Lonardi C, Cerutti R, Callus E, Niero M (2013). Gender-related Effect of Cultural Participation in Psychological Well-being: Indications from the Well-being Project in the Municipality of Milan. Soc Indic Res.

[59] Cocozza S, Sacco PL, Matarese G, Maffulli GD, Maffulli N, Tramontano D (2020). Participation to Leisure Activities and Well-Being in a Group of Residents of Naples-Italy: The Role of Resilience. Int J Environ Res Public Health.

[60] Bai X, Nath I, Capon A, Nordin Hasan N, Jaron D (2012). Health and wellbeing in the changing urban environment: complex challenges, scientific responses, and the way forward. Current Opinion in Environmental Sustainability.

[61] Næss Petter (2014). Urban Form, Sustainability and Health:The Case of Greater Oslo. European Planning Studies.

[62] VanderWeele TJ, Hawkley LC, Cacioppo JT (2012). On the reciprocal association between loneliness and subjective well-being. American Journal of Epidemiology.

[63] Holt-Lunstad J, Smith TB, Baker M, Harris T, Stephenson D (2015). Loneliness and social isolation as risk factors for mortality: a meta-analytic review. Perspect Psychol Sci.

[64] Steptoe A, Shankar A, Demakakos P, Wardle J (2013). Social isolation, loneliness, and all-cause mortality in older men and women. Proc Natl Acad Sci U S A.

[b65-tmj-23-04-092] 65 https://www.oecd.org/statistics/oecd-guidelines-on-measuring-subjective-well-being-9789264191655-en.htm

[66] Michaelson J, Seaford C, Abdallah S, Marks N, Huppert F, Cooper C (2013). Measuring what matters. Interventions and policies to enhance well-being.

[67] Sallis JF, Saelens BE (2000). Assessment of physical activity by self-report: status, limitations, and future directions. Res Q Exerc Sport.

[68] O’Laughlin KD, Martin MJ, Ferrer E (2018). Cross-Sectional Analysis of Longitudinal Mediation Processes. Multivar Behav Res.

[69] Patrick E, Shrout PE (2011). Commentary: Mediation Analysis, Causal Process, and Cross-Sectional Data. Multivar Behav Res.

[70] Rubin M, Kelly BM (2015). A cross-sectional investigation of parenting style and friendship as mediators of therelation between social class and mental health in a university community. Int J Equity Health.

[71] Pek J, Hoyle RH (2016). On the (In) Validity of Tests of Simple Mediation: Threats and Solutions. Soc Personal Psychol Compass.

